# Angiogenesis Dysregulation in Psoriatic Arthritis: Molecular Mechanisms

**DOI:** 10.1155/2017/5312813

**Published:** 2017-07-19

**Authors:** Francesco Paolo Cantatore, Nicola Maruotti, Addolorata Corrado, Domenico Ribatti

**Affiliations:** ^1^Rheumatology Clinic, Department of Medical and Surgical Sciences, University of Foggia Medical School, Foggia, Italy; ^2^Department of Basic Medical Sciences, Neurosciences and Sensory Organs, University of Bari Medical School, Bari, Italy; ^3^National Cancer Institute “Giovanni Paolo II”, Bari, Italy

## Abstract

There is evidence that psoriatic arthritis is closely linked to angiogenesis. Morphological changes described in blood vessels of psoriatic arthritis joints suggest the presence of a dysregulated angiogenesis resulting in the formation of immature vessels. Even if the reason of this inefficient angiogenesis is still unclear, an imbalance between angiogenic and antiangiogenic factors is probably responsible for inducing a dysregulated angiogenesis in psoriatic arthritis, which seems to be involved in its pathogenesis and clinical features. Nevertheless, among chronic arthritides, while angiogenesis in rheumatoid arthritis has been largely studied with a great amount of literature data, limited data on angiogenesis role in psoriatic arthritis are available. This review article is focused on current knowledge on the mechanisms responsible for dysregulated angiogenesis in psoriatic arthritis.

## 1. Introduction

Psoriatic arthritis (PsA) is a chronic arthritis, associated with psoriasis, classified with seronegative spondyloarthritis. It is characterized by involvement of metacarpophalangeal and interphalangeal joints of the hands and feet, as well as ankles and knees, often with extra-articular involvement, including eye and/or bowel involvement, sometimes with sacroiliac joints and/or spinal involvement. Joint lesions in PsA are characterized by an erosive arthritis with periosteal reactions occurring in enthesitis and syndesmophytes occurring in spondylitis. In particular, enthesitis and dactylitis are distinctive clinical features of PsA. As demonstrated by ultrasonography and magnetic resonance imaging, enthesis may be the first site involved by inflammation in PsA [1]. Other PsA distinctive features are the absence of serological tests for rheumatoid factor and anti-cyclic citrullinated peptide (CCP) antibodies. PsA is often associated with HLA-B27 in patients with axial involvement [2]. Although the global features of synovitis are not different between rheumatoid arthritis (RA) and PsA, synovium inflammation in PsA is characterized by more intense hypervascularity and infiltration of polymorphonuclear leukocytes [3, 4]. Even if PsA pathogenesis is still unclear, angiogenesis plays a crucial role in the early events in PsA. This review article is focused on the analysis of the current knowledge on the mechanisms responsible for angiogenesis dysregulation occurring in PsA.

## 2. Angiogenesis

Angiogenesis, the formation of new capillaries from preexisting vessels, plays an important role in synovitis pathogenesis. Angiogenesis begins with the production of angiogenic factors which are responsible for the activation of endothelial cells, which, in turn, secrete proteolytic enzymes such as matrix metalloproteinases (MMPs) and plasminogen activators. These enzymes degrade the basement membrane and the perivascular extracellular matrix. Subsequently, endothelial cells proliferate and migrate into the perivascular area. New vessel formation is then completed by lumenation of these “primary sprouts” forming “capillary loops” and by the synthesis of a new basement membrane. The proliferation of endothelial cells of these “primary sprouts” and their migration lead to the generation of secondary and further generations of vascular sprouts [5].

The regulation of these events is due to the net balance between angiogenic and antiangiogenic factors [6]. Vascular endothelial growth factor (VEGF) and fibroblast growth factor (FGF) family members, platelet-derived growth factor (PDGF), tumor necrosis factor-alpha (TNF-*α*), transforming growth factor-alpha and transforming growth factor-beta (TGF-*α* and TGF-*β*), interleukins (ILs), chemokines, angiogenin, and angiopoietins (Angs) are the main angiogenic factors. On the other hand, angiostatin, endostatin, and thrombospondin inhibit angiogenesis [7–9]. An imbalance between these positive and negative factors, with a predominance of angiogenic factors, or downregulation of inhibitory regulators, is involved in impaired angiogenesis which has been observed in several autoimmune diseases [6, 10, 11].

Angiogenesis appears to be a first-order event in psoriatic arthritis, as in RA. Alterations in the vascular morphology of the nail folds of patients affected by psoriasis without nail disease have been seen by microscopic examination [12], as well as an increase in the number of blood vessels and morphological vascular alterations, such as tortuous and elongated blood vessels, which have been observed in the PsA synovial membrane [13, 14].

Morphological vascular alterations in PsA synovial tissue appear to be manifestly distinct from that observed in RA. In fact, PsA is mainly characterized by tortuous, bushy, elongated vessels, whereas RA is prevalently characterized by straight vessels with regular branching [14]. This suggests that angiogenic pathways are different between PsA and RA, just like the pathogenic mechanisms. The morphological chances described in blood vessels of PsA joints are also observed in psoriatic skin lesions and suggest the presence of a dysregulated angiogenesis resulting in immature vessels [15]. Nevertheless, while angiogenesis in RA has been largely studied and a great amount of data is present in the literature [10, 16], there are only limited data on the role of angiogenesis in PsA.

## 3. The Role of Hypoxia

The role of hypoxia has been extensively studied in RA, while few data are available in PsA. Hypoxia in the rheumatoid joint has been demonstrated many years ago by direct measurements on synovial fluid samples from RA patients [17, 18]. Three mechanisms have been suggested to explain hypoxia in RA synovial tissue: (1) the intermittent closing of capillaries for the increased intra-articular pressure due to synovial hyperplasia, synovial fluid effusion, and joint movements within the rigid joint capsule; (2) the high metabolic demand due to the migration and proliferation of inflammatory cells, with an increment of the distance between proliferating cells and nearby blood vessels; (3) the increased expression of angiotensin converting enzyme (ACE) that induces the formation of angiotensin II which is responsible for vasoconstriction and enhancing hypoxia [19]. It is conceivable that the first two mechanisms may also be involved in PsA, while no data are available about ACE expression in PsA.

In PsA, low in vivo oxygen levels have been demonstrated in PsA synovium [20]. Hypoxia is involved in inducing the expression of angiogenic chemokines, MMPs, and hypoxia inducible factor (HIF) [21–25]. Expression of HIF-1*α* by macrophages has been observed principally close to the intimal layer but also in the subintimal area in rheumatoid synovium [26]. HIF induces VEGF transcription via hypoxia response element (HRE) interaction in the promoter of VEGF gene and is recognized as a key event in angiogenesis induction [27]. HIF-1*α* subunit stability is regulated by oxygen levels through the enzyme prolyl hydroxylase (PHD) [28]. Hypoxia is also responsible for inducing the expression of nuclear factor-*κ*B (NF-*κ*B) via decreased PHD-dependent hydroxylation of inhibitor of *κ*B kinase *β* (IKK*β*) [29].

Hypoxia induces the formation of reactive oxygen species (ROS), via activation of cellular systems, such as the mitochondrial electron transport chain and NADPH oxidase (NOX). ROS are responsible for oxidative damage that modifies the structure of DNA, proteins, and lipids and are involved in angiogenesis, endothelial cell differentiation, proliferation, and migration [30–33]. A significantly increased expression of NOX-2, which is the membrane-bound catalytic subunit of NOX, has been observed in a study on fifty-four patients with active inflammatory arthritis (33 with RA and 21 with PsA) [34]. High NOX-2 expression was correlated with low synovial PO_2_ levels and with high expression of VEGF, Ang-2, factor VIII, neural cell adhesion molecule, and *α*-smooth muscle actin. Moreover, a decrease in NOX-2 expression and an increase in in vivo synovial PO_2_ levels have been found in patients treated with anti-TNF-*α* [34].

Hypoxia has been correlated to altered bioenergetic and increased metabolic turnover in inflamed joints [35]. In fact, hypoxia is involved in mitochondrial dysfunction and induces a switch to glycolysis, supporting abnormal angiogenesis. Hypoxia and increased glycolytic metabolites are responsible for the expression of HIF-1*α* and NF-*κ*B. These transcription factors induce the expression of angiogenic growth factors, inflammatory cytokines, and extracellular membrane components which in turn are involved in further glycolysis [36].

## 4. Angiogenic Factors in PSA

Increased levels of several angiogenic factors, such as VEGF, TGF-*β*, PDGF, and Angs, have been found in psoriasis [37, 38]. Increasing evidence underlines the importance of numerous angiogenic factors also in PsA (Table 1). Angs and VEGF expression has been demonstrated in perivascular areas of PsA synovial membrane and increased expression of VEGF and TGF-*β* has been found in the synovial fluid in early PsA [15, 39]. Ang-2 and VEGF expression in the synovial membrane has been found to be significantly higher in early PsA than in RA. Moreover, significantly higher VEGF and TGF-*β* levels have been seen in the synovial fluid in early PsA compared to RA [40]. MMP-1 and MMP-3 have been described in the sublining and lining layer cells in the synovial tissue in PsA [41]. Moreover, MMP-9 levels have been found to be significantly higher in the synovial fluids of early PsA than in early RA. In the synovial membrane, MMP-9 levels have also been found to be higher in early PsA than in early RA, but without a significant difference [42].

Synovial vascular morphology appears to be related to angiogenic factors, such as VEGF, Angs, and MMP-9. In PsA, distinct vascular morphology, characterized by tortuous vessels, has been correlated with VEGF levels [40]. Moreover, in PsA lining layer hyperplasia is less evident than in RA, probably due to impaired apoptosis of lining cells and decreased presence of CD68+ macrophage-like synoviocytes [43].

The concomitant expression of these angiogenic molecules in PsA joints plays a key role in angiogenesis induction, as demonstrated by the more intense activation of an important angiogenic signaling pathway, the NOTCH-DLL4, after stimulation of VEGF and Ang-2 in combination compared with either VEGF or Ang-2 alone [44].

Accumulated evidence shows that PDGF has a key role in the recruitment of pericytes to newly formed vessels, where their primary function is to maintain the vessel integrity. In inflammatory arthritis, both immature and mature blood vessels have been found in the synovium. The presence of immature vessels may be responsible for instability of endothelial–pericyte interactions [45].

VEGF and its receptors, VEGFR-1/Flt1 and VEGFR-2/KDR, have been demonstrated in PsA synovial tissue, suggesting VEGF's role in inducing angiogenesis and vascular permeability [39, 46]. In the synovial tissue, VEGF may be derived from endothelial and synovial cells. Synovial VEGF levels may be upregulated by cytokines, such as IL-1 and TNF-*α*, secreted by inflammatory cells and synoviocytes [46]. Increased levels of VEGF have also been observed in serum obtained from PsA patients [47], produced by macrophages, fibroblasts, neutrophils, and platelets [48, 49]. Nevertheless, Przepiera-Będzak et al. [50] have found comparable VEGF, EGF, and FGF-2 serum levels in PsA patients and controls, even if the authors admit the presence of several limitations in their study including a small number of patients and no group of patients without treatment. In the same study, serum VEGF levels correlated with serological and clinical indicators, such as CRP and BASFI (Bath Ankylosing Spondylitis Functional Index), and disease duration [50]. VEGF polymorphisms have been associated with the onset of psoriasis [51, 52]. A low expression of the T allele of VEGF rs3025039, known as +936 C/T, has been found in PsA patients when compared with controls [53]. This suggests that this polymorphism has a protective role against the development of PsA. It is interesting to underline that the frequency of the 936 T allele is significantly increased in RA patients, suggesting that different mechanisms are involved in angiogenesis in PsA and RA [54].

Inflammatory cytokines involved in PsA pathogenesis, such as TNF-*α*, also have angiogenic effects. TNF-*α* is involved in the induction and upregulation of angiogenic agents, such as VEGF [15]. A similar role is also conceivable for IL-1 [15]. In synovial fibroblasts, TNF-*α*, via stimulation of Toll-like receptor-2 (TLR-2) pathway, induces the translocation of NF-*κ*B, which is responsible for inducing the expression of proinflammatory cytokines and MMPs [46, 55]. IL-17, produced by T-helper-17 via stimulation of IL-23, induces upregulation of proinflammatory cytokines, neutrophils chemiotaxis, endothelial cell migration [46], suggesting a role for IL-17 and IL-23 in angiogenesis in psoriatic arthritis. Oncostatin M (OSM) is a member of the IL-6 family, which has a role in arthritis pathogenesis [56]. Its involvement in angiogenesis has been seen in RA [57]. More recently, a role for OSM has been described in IL-17 regulation [58]. Thus, it is conceivable that OSM may play a role in PsA angiogenesis.

Therapies with TNF-*α* inhibitors have been associated with reduced levels of VEGF in sera and skin of patients affected by PsA [59, 60]. Immunohistochemical studies on synovial and psoriatic lesional skin biopsies obtained from PsA patients treated with anti-TNF-*α* agents have demonstrated changes in numerous factors involved in angiogenesis regulation [61, 62]. Lower levels of VEGF and VEGFR-2 and a reduced expression of stromal cell-derived factor 1 (SDF1)/CXC motif chemokine 12 (CXCL12) and Tie2 have been found in PsA patients treated with anti-TNF-*α* agents [61]. Tie2 is a specific receptor for Ang-1 and Ang-2. The Angs and Tie-2 are also important regulators of blood vessel growth, maturation, and function. Ang-1 is characterized by angiogenic effects, whereas Ang-2 has generally an opposing action [63]. Ang-2 levels increased after anti-TNF-*α* therapy [61]. By considering that Ang-2 acts as both a Tie-2 antagonist and agonist [64], it is conceivable that Ang-2 induces angiogenesis in the presence of VEGF, whereas it is involved in vascular regression when VEGF is downregulated [65]. As suggested by Cañete et al. [61], an increase in the Ang-2/VEGF ratio after anti-TNF-*α* treatment, as well as Tie2 reduction, may be responsible for the consistent reduction of synovial neovascularization in PsA. By considering that studies have shown a synergistic effect of TNF-*α* and Angs in driving inflammation and angiogenesis [66], it is conceivable that this could have a role in partial responders to anti-TNF-*α* treatment.

Finally, a significant reduction in the expression of MMP-9 and adhesion molecules, such as *α*v*β*3 integrin E-selectin, intercellular adhesion molecule-1 (ICAM-1), and vascular cell adhesion molecule-1 (VCAM-1), and in the number of blood vessels in dermis and/or synovium has been demonstrated after anti-TNF-*α* therapy [62, 67].

## 5. Concluding Remarks

Alterations in blood vessels' morphology in joints and psoriatic skin lesions suggest the presence of a dysregulated angiogenesis resulting in immature vessels in PsA [15]. Pathogenic mechanisms of this inefficient angiogenesis in PsA are still unclear. However, an imbalance between angiogenic and antiangiogenic factors is probably involved in inducing a dysregulated angiogenesis in PsA, which seems to play an important role in its pathogenesis and clinical implications. Further studies are needed to explain the role of angiogenesis in the pathogenesis of PsA and to clarify the mechanism responsible for angiogenesis dysregulation.

## Figures and Tables

**Table 1:** Angiogenic agents involved in PsA.

Angiogenic factors in PsA	
VEGF	[40, 47, 61]
TGF-*β*	[40]
Ang-1	[40]
Ang-2 (both stimulator and inhibitor)	[40, 61]
MMP-9	[42]
TNF-*α*	[59–62, 67]

VEGF: vascular endothelial growth factor; TGF-*β*: transforming growth factor-*β*; Ang: angiopoietin; MMP-9: matrix metalloproteinase-9; TNF-*α*: tumor necrosis factor-*α*.
